# Supplementary material for: OpenMM 7: Rapid development of high performance algorithms for molecular dynamics
Source: PLoS Comput Biol. 2017 Jul 26;13(7):e1005659. doi: 10.1371/journal.pcbi.1005659 (PMC5549999; doi:10.1371/journal.pcbi.1005659)
Supplement: S1 Source Code — (ZIP) [file pcbi.1005659.s002.zip › OpenMM-7.0.1-Source/docs/api-python/generated/simtk.openmm.openmm.XmlSerializer.html]

XmlSerializer — OpenMM 7.0.1.dev-5e86c4f documentation


# XmlSerializer¶

*class* `simtk.openmm.openmm.``XmlSerializer`(*\*args*, *\*\*kwargs*)¶
:   XmlSerializer is used for serializing objects as XML, and for reconstructing them again.

    `__init__`(*\*args*, *\*\*kwargs*)¶

    Methods

    |  |  |
    | --- | --- |
    | `__init__`(\*args, \*\*kwargs) |  |
    | `deserialize`(inputString) | Reconstruct an object that has been serialized as XML. |
    | `deserializeSystem`((inputString) -> System) | This method exists only for backward compatibility. |
    | `serialize`(object) | Serialize an object as XML. |
    | `serializeSystem`((object) -> std::string) | This method exists only for backward compatibility. |

    *static* `serializeSystem`(*object*) → std::string¶
    :   This method exists only for backward compatibility. @deprecated Use serialize() instead.

    *static* `deserializeSystem`(*inputString*) → System¶
    :   This method exists only for backward compatibility. @deprecated Use deserialize() instead.

    *static* `serialize`(*object*)¶
    :   Serialize an object as XML.

    *static* `deserialize`(*inputString*)¶
    :   Reconstruct an object that has been serialized as XML.

    `__delattr__`¶
    :   x.\_\_delattr\_\_(‘name’) <==> del x.name

    `__format__`()¶
    :   default object formatter

    `__getattribute__`¶
    :   x.\_\_getattribute\_\_(‘name’) <==> x.name

    `__hash__`¶

    `__reduce__`()¶
    :   helper for pickle

    `__reduce_ex__`()¶
    :   helper for pickle

    `__sizeof__`() → int¶
    :   size of object in memory, in bytes

    `__str__`¶

High performance molecular simulation on GPUs


### Navigation

- Application Layer
- Library Layer
  - Core Objects
  - Forces
  - Integrators
  - Extras

©2015, Stanford University and the Authors.
|
Powered by Sphinx 1.3.1
& Alabaster 0.7.7
|
Page source
